# Bis[(1,10-phenanthroline-κ^2^ *N*,*N*′)bis­(triphenyl­phosphane-κ*P*)copper(I)] nona­deca­oxidohexa­molybdate(VI)

**DOI:** 10.1107/S1600536812036367

**Published:** 2012-08-25

**Authors:** Fabienne Gschwind, Martin Jansen

**Affiliations:** a Max Planck Institute for Solid State Research, Heisenbergstrasse 1, 70569 Stuttgart, Germany

## Abstract

The title compound, [Cu(C_12_H_8_N_2_)(C_18_H_15_P)_2_]_2_[Mo_6_O_19_], was obtained by co-crystallization of the mixed-ligand copper complex cation (1,10-phenanthroline)bis­(triphenyl­phos­phane)copper(I), [Cu(phen)(PPh_3_)_2_]^+^, with the Lindquist polyanion [Mo_6_O_19_]^2−^. The asymmetric unit consists of half a Lindquist anion and one [Cu(phen)(PPh_3_)_2_]^+^ cationic complex. In the cation, there are intra­molecular π–π inter­actions [centroid–centroid distances = 3.617 (2) and 3.7272 (18) Å]. This inorganic–organic adduct is connected by C—H⋯O hydrogen bonds, forming a two dimensional network lying in the *ab* plane. These networks are connected by C—H⋯π inter­actions into a three-dimensional structure.

## Related literature

For general background to mixed-ligand copper complexes and Lindquist anions, see: Gruber & Jansen (2011 ▶). For details of the [Mo_6_O_19_]^2−^ polyoxido­anion, see: Jaypal *et al.* (2010 ▶); Rheingold *et al.* (1993 ▶). For the synthesis of the (1,10-phen­an­throline)bis­(triphenyl­phosphane)copper(I) complex cation [Cu(phen)(PPh_3_)_2_]^+^, see: McMillin *et al.* (1985 ▶). For the synthesis of polyoxidoanions and the Anderson-type heteropolyanion [Al(OH)_6_Mo_6_O_18_], see: Kemperer & Silavwe (2007 ▶). For examples of combinations of Lindquist anions with copper(I) complexes, see: Sha *et al.* (2009 ▶); Hou *et al.* (2011 ▶). For the synthesis of Cu^2+^ complexes, see: Shivaiah *et al.* (2007 ▶).
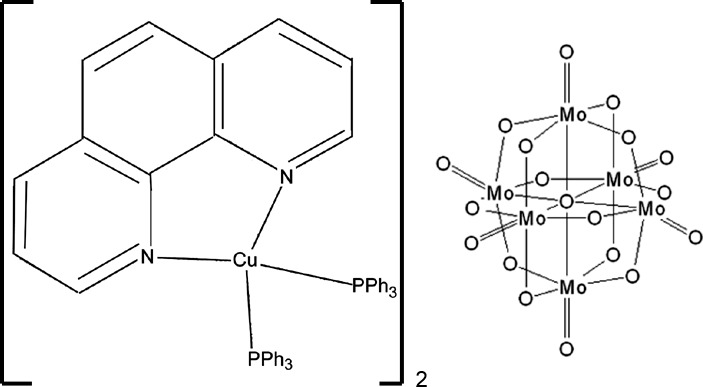


## Experimental

### 

#### Crystal data

[Cu(C_12_H_8_N_2_)(C_18_H_15_P)_2_]_2_[Mo_6_O_19_]
*M* *_r_* = 2416.23
Triclinic, 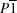

*a* = 11.287 (2) Å
*b* = 13.572 (3) Å
*c* = 16.307 (3) Å
α = 109.03 (3)°
β = 102.98 (3)°
γ = 93.93 (3)°
*V* = 2273.7 (8) Å^3^
*Z* = 1
Mo *K*α radiation
μ = 1.40 mm^−1^
*T* = 298 K
0.3 × 0.2 × 0.1 mm

#### Data collection

Stoe IPDS 2 diffractometer
Absorption correction: numerical (*X-SHAPE*; Stoe & Cie, 2009 ▶) *T*_min_ = 0.818, *T*_max_ = 0.908
22882 measured reflections
9593 independent reflections
7714 reflections with *I* > 2σ(*I*)
*R*_int_ = 0.024

#### Refinement

*R*[*F* ^2^ > 2σ(*F* ^2^)] = 0.026
*wR*(*F* ^2^) = 0.063
*S* = 0.86
9593 reflections
592 parameters
H-atom parameters constrained
Δρ_max_ = 0.25 e Å^−3^
Δρ_min_ = −0.57 e Å^−3^

### 

Data collection: *X-AREA* (Stoe & Cie, 2009 ▶); cell refinement: *X-AREA*; data reduction: *X-RED32* (Stoe & Cie, 2009 ▶); program(s) used to solve structure: *SHELXS97* (Sheldrick, 2008 ▶); program(s) used to refine structure: *SHELXL97* (Sheldrick, 2008 ▶); molecular graphics: *DIAMOND* (Brandenburg, 2006 ▶); software used to prepare material for publication: *publCIF* (Westrip, 2010 ▶).

## Supplementary Material

Crystal structure: contains datablock(s) I, global. DOI: 10.1107/S1600536812036367/hg5234sup1.cif

Structure factors: contains datablock(s) I. DOI: 10.1107/S1600536812036367/hg5234Isup2.hkl

Additional supplementary materials:  crystallographic information; 3D view; checkCIF report

## Figures and Tables

**Table 1:** Hydrogen-bond geometry (Å, °) *Cg*8 is the centroid of the C33–C38 ring.

*D*—H⋯*A*	*D*—H	H⋯*A*	*D*⋯*A*	*D*—H⋯*A*
C19—H19⋯O3^i^	0.93	2.60	3.446 (3)	152
C29—H29⋯O7^ii^	0.93	2.55	3.443 (4)	161
C30—H30*A*⋯O8^iii^	0.93	2.41	3.257 (5)	152
C44—H44⋯O2	0.93	2.51	3.206 (4)	132
C49—H49⋯*Cg*8^iv^	0.93	2.97	3.782 (5)	147

Symmetry codes: (i) 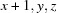; (ii) 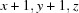; (iii) 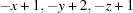; (iv) 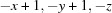.

**Fig. 1.**
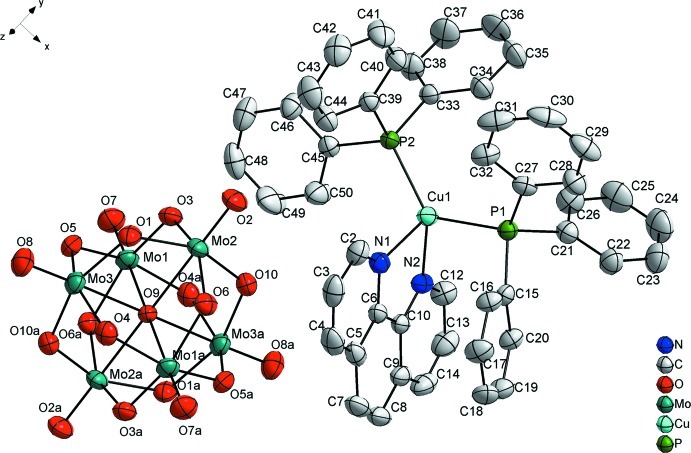
A view of the molecular structure of the title compound, with the atom numbering. The displacement ellipsoids are drawn at the 50% probability level. Symmetry code: (a) -x + 1, -y + 1, -z + 1.

**Fig. 2.**
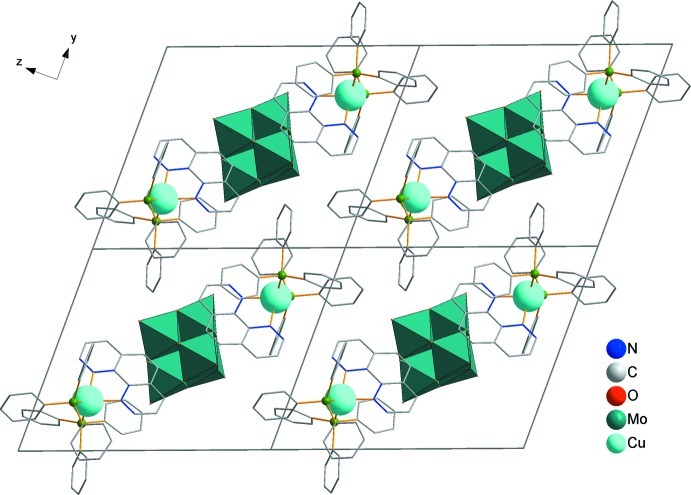
A view along the x-axis of the crystal packing of the title compound.

## supplementary crystallographic information

### Comment

Only very few examples of combinations between Lindquist anions with copper(I) complexes exist. Some examples are the MOF-like honeycomb compounds of (Sha *et al.*, 2009*)* and the charged directed assemblies of (Hou *et al.*, 2011) which form macrocycles and polymer chains. Another example is the Anderson-type heteropolyanion (Al(OH)_6_Mo_6_O_18_) connected to a copper(I)phenanthroline complex which has been investigated by (Shivaiah *et al.*, 2007) and possesess large water-pipes formed by 28 cocrystallized water molecules.

The mixed-ligand copper complex (1,10-phenanthroline)bis(triphenylphosphanecopper(I), [Cu(phen)(PPh_3_)_2_]^+^, was cocrystallized with the Lindquist polyanion [Mo_6_O_19_]^2-^ to form the title compound (Fig. 1). The asymmetric unit consists of half a Lindquist anion, and one [Cu(phen)(PPh_3_)_2_]^+^ cationic complex.

In the cation there are two intramolecular π–π contacts present: one involving ring N1/C2–C6 (*Cg*(2)) of the phenanthroline moiety and phenyl ring C15–C20 (*Cg*(5)) with a centroid–centroid distance of 3.7272 (18) Å; the other involves ring C5–C10 (*Cg*(4)) of the phenanthroline moiety and the same phenyl ring C15–C20 (*Cg*(5)) with a centroid-centroid distance of 3.617 (2) Å.

In the crystal, each [Cu(phen)(PPh_3_)_2_]^+^ cation connects *via* C—H···O hydrogen bonds (Table 1 and Fig. 2) to neighbouring Linquist anions, [Mo_6_O_19_]^2-^, to form a two-dimensional hydrogen bonded network lying in the *ab* plane.

These networks are linked *via* C—H···π interactions to form a densely packed three-dimensional structure (Table 1).

The polyoxoanion [Mo_6_O_19_]^2-^ is built from six distorted MoO_6_ octahedra sharing common edges and one common vertex at the central O atom, and has crystallographic *m*3*m* (Oh) symmetry (Jaypal *et al.*, 2010; Rheingold *et al.*, 1993). The commercially available complex (1,10-phenanthroline)bis(triphenylphosphanecopper(I), [Cu(phen)(PPh_3_)_2_]^+^, was first characterized by McMillin *et al.*, ((1985). In our case the coordination geometry (distorted tetrahedral) and the average Cu—N [2.098 (2) Å] and Cu—P [2.2599 (4) Å] distances fit very well to the values for the free complexes [Cu—N 2.075 Å and Cu—P 2.258 Å]. This shows that the building blocks retain their original conformations (Gruber & Jansen, 2011).

### Experimental

The polyoxomolybdate [NBu_4_]_2_[Mo_6_O_19_] was prepared following the synthetic procedure of tetrabutylammonium hexatungstate, replacing sodium tungstate with sodium molybdate (Kemperer & Silavwe, 2007). The title compound was prepared by mixing 0.24 g of (1,10- phenanthroline)bis(triphenylphosphanecopper(I) and the 0.2 g polymolybdate dissolved in a 1:1 mixture of acetonitrile (15 ml) and methanol (15 ml), and stirred over–night. The reaction-solution was overlayered with diethylether. Green crystals, suitable for X-ray diffraction analysis, grew after a few days at the interface of the solvents.

### Refinement

The C-bound H-atoms were included in calculated positions and treated as riding atoms: C—H = 0.93 Å with *U*_iso_(H) = 1.2*U*_eq_(parent C-atom).

### Crystal data

**Table:**

[Cu(C_12_H_8_N_2_)(C_18_H_15_P)_2_]_2_[Mo_6_O_19_]	*Z* = 1
*M**_r_* = 2416.23	*F*(000) = 1202
Triclinic, *P*1	*D*_x_ = 1.765 Mg m^−^^3^
Hall symbol: -P 1	Mo *K*α radiation, λ = 0.71073 Å
*a* = 11.287 (2) Å	Cell parameters from 22955 reflections
*b* = 13.572 (3) Å	θ = 1.3–26.7°
*c* = 16.307 (3) Å	µ = 1.40 mm^−^^1^
α = 109.03 (3)°	*T* = 298 K
β = 102.98 (3)°	Block, green
γ = 93.93 (3)°	0.3 × 0.2 × 0.1 mm
*V* = 2273.7 (8) Å^3^	

### Data collection

**Table:**

Stoe IPDS 2 diffractometer	9593 independent reflections
Radiation source: fine-focus sealed tube	7714 reflections with *I* > 2σ(*I*)
Graphite monochromator	*R*_int_ = 0.024
Detector resolution: 6.67 pixels mm^-1^	θ_max_ = 26.7°, θ_min_ = 1.4°
ω and φ scans	*h* = −14→14
Absorption correction: numerical (*X-SHAPE*; Stoe & Cie, 2009)	*k* = −17→17
*T*_min_ = 0.818, *T*_max_ = 0.908	*l* = −19→20
22882 measured reflections	

### Refinement

**Table:**

Refinement on *F*^2^	Primary atom site location: structure-invariant direct methods
Least-squares matrix: full	Secondary atom site location: difference Fourier map
*R*[*F*^2^ > 2σ(*F*^2^)] = 0.026	Hydrogen site location: inferred from neighbouring sites
*wR*(*F*^2^) = 0.063	H-atom parameters constrained
*S* = 0.86	*w* = 1/[σ^2^(*F*_o_^2^) + (0.0449*P*)^2^] where *P* = (*F*_o_^2^ + 2*F*_c_^2^)/3
9593 reflections	(Δ/σ)_max_ = 0.001
592 parameters	Δρ_max_ = 0.25 e Å^−^^3^
0 restraints	Δρ_min_ = −0.57 e Å^−^^3^

### Special details

**Table:**

Geometry. All e.s.d.'s (except the e.s.d. in the dihedral angle between two l.s. planes) are estimated using the full covariance matrix. The cell e.s.d.'s are taken into account individually in the estimation of e.s.d.'s in distances, angles and torsion angles; correlations between e.s.d.'s in cell parameters are only used when they are defined by crystal symmetry. An approximate (isotropic) treatment of cell e.s.d.'s is used for estimating e.s.d.'s involving l.s. planes.
Refinement. Refinement of *F*^2^ against ALL reflections. The weighted *R*-factor *wR* and goodness of fit *S* are based on *F*^2^, conventional *R*-factors *R* are based on *F*, with *F* set to zero for negative *F*^2^. The threshold expression of *F*^2^ > σ(*F*^2^) is used only for calculating *R*-factors(gt) *etc*. and is not relevant to the choice of reflections for refinement. *R*-factors based on *F*^2^ are statistically about twice as large as those based on *F*, and *R*- factors based on ALL data will be even larger.

### Fractional atomic coordinates and isotropic or equivalent isotropic displacement parameters (Å^2^)

**Table:**

	*x*	*y*	*z*	*U*_iso_*/*U*_eq_	
N1	0.77376 (18)	0.75215 (17)	0.33346 (13)	0.0316 (4)	
C2	0.7543 (2)	0.8190 (2)	0.40689 (18)	0.0395 (6)	
H2	0.7061	0.8704	0.4011	0.047*	
C3	0.8023 (3)	0.8165 (3)	0.49267 (18)	0.0475 (7)	
H3	0.7852	0.8646	0.5424	0.057*	
C4	0.8742 (3)	0.7429 (3)	0.50257 (19)	0.0496 (7)	
H4	0.9069	0.7403	0.5594	0.060*	
C5	0.8988 (2)	0.6712 (2)	0.42729 (18)	0.0392 (6)	
C6	0.8446 (2)	0.6764 (2)	0.34301 (16)	0.0306 (5)	
C7	0.9768 (3)	0.5924 (3)	0.4315 (2)	0.0490 (7)	
H7	1.0152	0.5893	0.4872	0.059*	
C8	0.9951 (2)	0.5240 (3)	0.3575 (2)	0.0478 (7)	
H8	1.0459	0.4740	0.3627	0.057*	
C9	0.9388 (2)	0.5253 (2)	0.27000 (19)	0.0371 (6)	
C10	0.86382 (19)	0.60291 (19)	0.26322 (17)	0.0305 (5)	
N2	0.81050 (17)	0.61224 (16)	0.18323 (13)	0.0310 (4)	
C12	0.8292 (2)	0.5449 (2)	0.10935 (18)	0.0394 (6)	
H12	0.7927	0.5507	0.0543	0.047*	
C13	0.9012 (3)	0.4656 (2)	0.1106 (2)	0.0480 (7)	
H13	0.9120	0.4195	0.0573	0.058*	
C14	0.9558 (2)	0.4563 (2)	0.1909 (2)	0.0473 (7)	
H14	1.0042	0.4038	0.1925	0.057*	
C15	1.0301 (2)	0.85331 (19)	0.29914 (16)	0.0294 (5)	
C16	1.0508 (2)	0.9196 (2)	0.38800 (18)	0.0382 (6)	
H16	1.0101	0.9778	0.4020	0.046*	
C17	1.1321 (3)	0.8991 (3)	0.45586 (18)	0.0459 (7)	
H17	1.1453	0.9434	0.5153	0.055*	
C18	1.1938 (2)	0.8131 (3)	0.4357 (2)	0.0463 (7)	
H18	1.2478	0.7995	0.4815	0.056*	
C19	1.1749 (2)	0.7481 (2)	0.3481 (2)	0.0430 (6)	
H19	1.2173	0.6909	0.3344	0.052*	
C20	1.0929 (2)	0.7674 (2)	0.28012 (17)	0.0351 (5)	
H20	1.0796	0.7223	0.2210	0.042*	
C21	0.9724 (2)	0.8439 (2)	0.11557 (17)	0.0358 (5)	
C22	1.0965 (3)	0.8739 (3)	0.12552 (19)	0.0454 (7)	
H22	1.1505	0.8999	0.1826	0.054*	
C23	1.1404 (3)	0.8655 (3)	0.0515 (2)	0.0592 (8)	
H23	1.2236	0.8861	0.0589	0.071*	
C24	1.0614 (4)	0.8270 (3)	−0.0332 (2)	0.0657 (10)	
H24	1.0911	0.8218	−0.0830	0.079*	
C25	0.9396 (4)	0.7963 (3)	−0.0442 (2)	0.0625 (9)	
H25	0.8862	0.7709	−0.1016	0.075*	
C26	0.8947 (3)	0.8027 (3)	0.02979 (19)	0.0475 (7)	
H26	0.8121	0.7791	0.0215	0.057*	
C27	0.8914 (2)	1.0043 (2)	0.24709 (16)	0.0331 (5)	
C28	0.9738 (3)	1.0803 (2)	0.2400 (2)	0.0453 (6)	
H28	1.0402	1.0607	0.2168	0.054*	
C33	0.4973 (2)	0.7708 (2)	0.03642 (16)	0.0320 (5)	
C29	0.9572 (3)	1.1848 (2)	0.2673 (2)	0.0566 (8)	
H29	1.0123	1.2349	0.2618	0.068*	
C34	0.5743 (3)	0.8412 (2)	0.01921 (19)	0.0441 (6)	
H34	0.6434	0.8814	0.0640	0.053*	
C35	0.5489 (3)	0.8519 (3)	−0.0645 (2)	0.0618 (9)	
H35	0.6004	0.8998	−0.0755	0.074*	
C30	0.8613 (3)	1.2152 (3)	0.3019 (2)	0.0606 (9)	
H30A	0.8511	1.2858	0.3200	0.073*	
C36	0.4480 (3)	0.7919 (3)	−0.1310 (2)	0.0676 (10)	
H36	0.4318	0.7982	−0.1873	0.081*	
C31	0.7794 (3)	1.1418 (3)	0.3103 (2)	0.0589 (9)	
H31A	0.7145	1.1629	0.3349	0.071*	
C32	0.7935 (3)	1.0358 (2)	0.28200 (19)	0.0442 (6)	
H32	0.7369	0.9860	0.2866	0.053*	
C37	0.3714 (3)	0.7229 (3)	−0.1144 (2)	0.0648 (10)	
H37	0.3025	0.6828	−0.1594	0.078*	
C38	0.3955 (3)	0.7122 (3)	−0.03103 (18)	0.0477 (7)	
H38	0.3426	0.6649	−0.0204	0.057*	
C39	0.4811 (2)	0.8713 (2)	0.21782 (16)	0.0307 (5)	
C40	0.4625 (3)	0.9616 (2)	0.19788 (19)	0.0430 (6)	
H40	0.4746	0.9656	0.1444	0.052*	
C41	0.4265 (3)	1.0456 (2)	0.2562 (2)	0.0556 (8)	
H41	0.4155	1.1060	0.2420	0.067*	
C42	0.4066 (3)	1.0410 (3)	0.3349 (2)	0.0550 (8)	
H42	0.3809	1.0973	0.3736	0.066*	
C43	0.4250 (3)	0.9524 (3)	0.3559 (2)	0.0568 (8)	
H43	0.4129	0.9492	0.4096	0.068*	
C44	0.4614 (3)	0.8676 (2)	0.29817 (18)	0.0426 (6)	
H44	0.4728	0.8078	0.3131	0.051*	
C45	0.4334 (2)	0.6472 (2)	0.14044 (16)	0.0317 (5)	
C46	0.3092 (2)	0.6507 (2)	0.1356 (2)	0.0431 (6)	
H46	0.2764	0.7109	0.1327	0.052*	
C47	0.2343 (3)	0.5654 (3)	0.1349 (2)	0.0532 (8)	
H47	0.1514	0.5685	0.1316	0.064*	
C48	0.2821 (3)	0.4760 (3)	0.1391 (2)	0.0565 (8)	
H48	0.2317	0.4190	0.1394	0.068*	
C49	0.4036 (3)	0.4711 (3)	0.1428 (3)	0.0678 (10)	
H49	0.4357	0.4104	0.1449	0.081*	
C50	0.4790 (3)	0.5563 (2)	0.1436 (2)	0.0525 (8)	
H50	0.5616	0.5523	0.1463	0.063*	
O1	0.38560 (15)	0.66417 (14)	0.55476 (12)	0.0365 (4)	
O2	0.48099 (18)	0.77098 (16)	0.45498 (14)	0.0468 (5)	
O3	0.35276 (15)	0.56076 (15)	0.38050 (12)	0.0370 (4)	
O4	0.53226 (16)	0.60361 (15)	0.67428 (11)	0.0366 (4)	
O5	0.25802 (14)	0.46973 (14)	0.47725 (12)	0.0352 (4)	
O6	0.62731 (15)	0.69308 (14)	0.57680 (12)	0.0352 (4)	
O7	0.20889 (17)	0.35874 (17)	0.28887 (13)	0.0474 (5)	
O8	0.27708 (17)	0.57790 (17)	0.66223 (14)	0.0477 (5)	
O10	0.59376 (15)	0.58943 (14)	0.40197 (12)	0.0361 (4)	
Mo1	0.332353 (18)	0.417707 (18)	0.376775 (14)	0.03313 (6)	
Mo2	0.488590 (19)	0.656614 (17)	0.473466 (15)	0.03361 (6)	
Mo3	0.368576 (19)	0.544685 (18)	0.592333 (15)	0.03407 (6)	
Cu1	0.73671 (2)	0.75378 (2)	0.202353 (19)	0.02976 (7)	
P1	0.90530 (5)	0.86401 (5)	0.21108 (4)	0.02878 (13)	
P2	0.53840 (5)	0.75954 (5)	0.14762 (4)	0.02702 (12)	
O9	0.5000	0.5000	0.5000	0.0240 (4)	

### Atomic displacement parameters (Å^2^)

**Table:**

	*U*^11^	*U*^22^	*U*^33^	*U*^12^	*U*^13^	*U*^23^
N1	0.0313 (10)	0.0342 (11)	0.0294 (10)	0.0039 (8)	0.0077 (8)	0.0118 (9)
C2	0.0360 (13)	0.0427 (16)	0.0377 (14)	0.0036 (11)	0.0126 (11)	0.0099 (12)
C3	0.0464 (15)	0.0571 (19)	0.0314 (13)	−0.0081 (14)	0.0135 (11)	0.0066 (13)
C4	0.0484 (16)	0.066 (2)	0.0330 (14)	−0.0054 (14)	0.0029 (12)	0.0240 (15)
C5	0.0351 (12)	0.0449 (16)	0.0383 (14)	−0.0057 (11)	0.0027 (10)	0.0224 (13)
C6	0.0249 (10)	0.0354 (13)	0.0345 (12)	0.0011 (9)	0.0050 (9)	0.0188 (11)
C7	0.0402 (14)	0.0559 (19)	0.0551 (18)	0.0020 (13)	−0.0038 (13)	0.0369 (16)
C8	0.0338 (13)	0.0505 (18)	0.069 (2)	0.0085 (12)	0.0044 (13)	0.0400 (17)
C9	0.0257 (11)	0.0339 (14)	0.0553 (16)	0.0032 (10)	0.0071 (10)	0.0230 (13)
C10	0.0230 (10)	0.0308 (13)	0.0409 (13)	0.0021 (9)	0.0058 (9)	0.0190 (11)
N2	0.0303 (10)	0.0294 (11)	0.0328 (10)	0.0066 (8)	0.0059 (8)	0.0115 (9)
C12	0.0384 (13)	0.0372 (15)	0.0368 (14)	0.0061 (11)	0.0071 (11)	0.0074 (12)
C13	0.0445 (15)	0.0383 (16)	0.0558 (18)	0.0094 (12)	0.0172 (13)	0.0060 (14)
C14	0.0347 (13)	0.0339 (15)	0.074 (2)	0.0113 (11)	0.0147 (13)	0.0190 (15)
C15	0.0231 (10)	0.0316 (13)	0.0339 (12)	0.0021 (9)	0.0070 (9)	0.0127 (11)
C16	0.0356 (12)	0.0372 (15)	0.0393 (14)	0.0118 (11)	0.0089 (11)	0.0094 (12)
C17	0.0436 (15)	0.0550 (19)	0.0315 (13)	0.0105 (13)	0.0037 (11)	0.0089 (13)
C18	0.0335 (13)	0.064 (2)	0.0471 (16)	0.0132 (12)	0.0036 (11)	0.0301 (15)
C19	0.0340 (13)	0.0459 (16)	0.0530 (17)	0.0149 (11)	0.0117 (12)	0.0204 (14)
C20	0.0316 (12)	0.0344 (14)	0.0368 (13)	0.0078 (10)	0.0087 (10)	0.0085 (11)
C21	0.0407 (13)	0.0346 (14)	0.0337 (13)	0.0088 (11)	0.0108 (10)	0.0128 (11)
C22	0.0414 (14)	0.0573 (19)	0.0399 (14)	0.0070 (13)	0.0126 (11)	0.0191 (14)
C23	0.0530 (17)	0.075 (2)	0.060 (2)	0.0114 (16)	0.0291 (15)	0.0281 (18)
C24	0.082 (2)	0.080 (3)	0.0453 (18)	0.017 (2)	0.0322 (17)	0.0247 (18)
C25	0.080 (2)	0.069 (2)	0.0313 (15)	0.0105 (18)	0.0099 (15)	0.0108 (16)
C26	0.0475 (15)	0.0504 (18)	0.0377 (14)	0.0052 (13)	0.0076 (12)	0.0093 (13)
C27	0.0358 (12)	0.0299 (13)	0.0304 (12)	0.0066 (10)	0.0017 (10)	0.0110 (11)
C28	0.0528 (16)	0.0360 (15)	0.0476 (16)	0.0035 (12)	0.0133 (13)	0.0161 (13)
C33	0.0321 (11)	0.0363 (14)	0.0312 (12)	0.0111 (10)	0.0099 (9)	0.0141 (11)
C29	0.078 (2)	0.0342 (16)	0.0512 (18)	−0.0013 (15)	0.0015 (16)	0.0190 (15)
C34	0.0427 (14)	0.0526 (18)	0.0419 (15)	0.0028 (12)	0.0075 (11)	0.0262 (14)
C35	0.064 (2)	0.081 (3)	0.0583 (19)	0.0035 (17)	0.0147 (16)	0.0494 (19)
C30	0.076 (2)	0.0313 (16)	0.058 (2)	0.0152 (15)	−0.0122 (17)	0.0125 (15)
C36	0.069 (2)	0.105 (3)	0.0437 (17)	0.019 (2)	0.0097 (15)	0.047 (2)
C31	0.0549 (18)	0.053 (2)	0.0558 (19)	0.0285 (16)	0.0021 (15)	0.0059 (16)
C32	0.0404 (14)	0.0419 (16)	0.0456 (15)	0.0120 (12)	0.0062 (12)	0.0117 (13)
C37	0.0557 (18)	0.096 (3)	0.0355 (15)	−0.0036 (18)	−0.0028 (13)	0.0262 (18)
C38	0.0447 (15)	0.060 (2)	0.0344 (14)	−0.0022 (13)	0.0054 (11)	0.0168 (14)
C39	0.0290 (11)	0.0315 (13)	0.0311 (12)	0.0033 (9)	0.0053 (9)	0.0123 (11)
C40	0.0542 (16)	0.0393 (16)	0.0455 (15)	0.0148 (12)	0.0218 (13)	0.0207 (13)
C41	0.072 (2)	0.0363 (17)	0.072 (2)	0.0204 (15)	0.0349 (17)	0.0246 (16)
C42	0.0656 (19)	0.0402 (17)	0.0587 (19)	0.0100 (14)	0.0318 (16)	0.0058 (15)
C43	0.077 (2)	0.053 (2)	0.0420 (16)	0.0074 (16)	0.0287 (15)	0.0110 (15)
C44	0.0550 (16)	0.0395 (16)	0.0379 (14)	0.0064 (12)	0.0153 (12)	0.0176 (13)
C45	0.0329 (11)	0.0312 (13)	0.0314 (12)	0.0030 (9)	0.0083 (9)	0.0119 (11)
C46	0.0391 (13)	0.0343 (15)	0.0542 (17)	0.0035 (11)	0.0169 (12)	0.0107 (13)
C47	0.0460 (16)	0.0453 (18)	0.0620 (19)	−0.0043 (13)	0.0279 (14)	0.0039 (15)
C48	0.071 (2)	0.0468 (19)	0.0502 (17)	−0.0146 (15)	0.0235 (15)	0.0147 (15)
C49	0.071 (2)	0.0416 (19)	0.099 (3)	0.0047 (16)	0.015 (2)	0.041 (2)
C50	0.0444 (15)	0.0394 (17)	0.078 (2)	0.0079 (12)	0.0130 (15)	0.0285 (16)
O1	0.0363 (9)	0.0307 (9)	0.0466 (10)	0.0124 (7)	0.0155 (8)	0.0146 (8)
O2	0.0502 (11)	0.0387 (11)	0.0603 (12)	0.0110 (9)	0.0116 (9)	0.0302 (10)
O3	0.0332 (9)	0.0413 (10)	0.0395 (9)	0.0089 (7)	0.0026 (7)	0.0220 (9)
O4	0.0388 (9)	0.0391 (10)	0.0303 (9)	0.0038 (8)	0.0095 (7)	0.0101 (8)
O5	0.0237 (8)	0.0408 (10)	0.0424 (10)	0.0056 (7)	0.0087 (7)	0.0161 (8)
O6	0.0347 (9)	0.0287 (9)	0.0401 (10)	0.0018 (7)	0.0059 (7)	0.0125 (8)
O7	0.0355 (9)	0.0545 (13)	0.0433 (11)	−0.0015 (8)	−0.0040 (8)	0.0168 (10)
O8	0.0446 (10)	0.0517 (13)	0.0515 (11)	0.0103 (9)	0.0255 (9)	0.0150 (10)
O10	0.0360 (9)	0.0391 (10)	0.0399 (10)	0.0061 (7)	0.0134 (7)	0.0202 (8)
Mo1	0.02695 (10)	0.03652 (13)	0.03166 (11)	0.00235 (8)	−0.00018 (8)	0.01217 (9)
Mo2	0.03471 (11)	0.02880 (12)	0.04289 (12)	0.00829 (8)	0.00839 (9)	0.02038 (10)
Mo3	0.03162 (11)	0.03736 (13)	0.03706 (12)	0.00855 (9)	0.01645 (9)	0.01242 (10)
Cu1	0.02732 (14)	0.03106 (16)	0.03130 (15)	0.00607 (11)	0.00420 (11)	0.01348 (13)
P1	0.0275 (3)	0.0278 (3)	0.0308 (3)	0.0047 (2)	0.0066 (2)	0.0107 (3)
P2	0.0253 (3)	0.0291 (3)	0.0286 (3)	0.0045 (2)	0.0053 (2)	0.0138 (3)
O9	0.0214 (10)	0.0259 (12)	0.0260 (11)	0.0051 (8)	0.0051 (8)	0.0112 (9)

### Geometric parameters (Å, º)

**Table:**

N1—C2	1.320 (3)	C35—C36	1.371 (5)
N1—C6	1.372 (3)	C35—H35	0.9300
N1—Cu1	2.092 (2)	C30—C31	1.372 (5)
C2—C3	1.394 (4)	C30—H30A	0.9300
C2—H2	0.9300	C36—C37	1.362 (5)
C3—C4	1.356 (5)	C36—H36	0.9300
C3—H3	0.9300	C31—C32	1.393 (4)
C4—C5	1.394 (4)	C31—H31A	0.9300
C4—H4	0.9300	C32—H32	0.9300
C5—C6	1.399 (3)	C37—C38	1.382 (4)
C5—C7	1.440 (4)	C37—H37	0.9300
C6—C10	1.430 (4)	C38—H38	0.9300
C7—C8	1.330 (5)	C39—C40	1.384 (4)
C7—H7	0.9300	C39—C44	1.393 (4)
C8—C9	1.431 (4)	C39—P2	1.834 (3)
C8—H8	0.9300	C40—C41	1.379 (4)
C9—C14	1.388 (4)	C40—H40	0.9300
C9—C10	1.412 (3)	C41—C42	1.373 (5)
C10—N2	1.358 (3)	C41—H41	0.9300
N2—C12	1.324 (3)	C42—C43	1.369 (5)
N2—Cu1	2.104 (2)	C42—H42	0.9300
C12—C13	1.395 (4)	C43—C44	1.382 (4)
C12—H12	0.9300	C43—H43	0.9300
C13—C14	1.366 (4)	C44—H44	0.9300
C13—H13	0.9300	C45—C50	1.381 (4)
C14—H14	0.9300	C45—C46	1.391 (4)
C15—C16	1.390 (4)	C45—P2	1.821 (3)
C15—C20	1.391 (3)	C46—C47	1.382 (4)
C15—P1	1.823 (2)	C46—H46	0.9300
C16—C17	1.387 (4)	C47—C48	1.377 (5)
C16—H16	0.9300	C47—H47	0.9300
C17—C18	1.385 (4)	C48—C49	1.366 (5)
C17—H17	0.9300	C48—H48	0.9300
C18—C19	1.371 (4)	C49—C50	1.383 (4)
C18—H18	0.9300	C49—H49	0.9300
C19—C20	1.385 (4)	C50—H50	0.9300
C19—H19	0.9300	O1—Mo3	1.9236 (18)
C20—H20	0.9300	O1—Mo2	1.9336 (18)
C21—C26	1.382 (4)	O2—Mo2	1.6791 (18)
C21—C22	1.389 (4)	O3—Mo1	1.9189 (18)
C21—P1	1.831 (3)	O3—Mo2	1.933 (2)
C22—C23	1.380 (4)	O4—Mo1^i^	1.8939 (18)
C22—H22	0.9300	O4—Mo3	1.9571 (19)
C23—C24	1.374 (5)	O5—Mo3	1.9086 (19)
C23—H23	0.9300	O5—Mo1	1.9574 (18)
C24—C25	1.363 (5)	O6—Mo2	1.9238 (18)
C24—H24	0.9300	O6—Mo1^i^	1.9293 (18)
C25—C26	1.391 (4)	O7—Mo1	1.6849 (19)
C25—H25	0.9300	O8—Mo3	1.6811 (19)
C26—H26	0.9300	O10—Mo2	1.9174 (19)
C27—C32	1.384 (4)	O10—Mo3^i^	1.9238 (18)
C27—C28	1.392 (4)	Mo1—O4^i^	1.8939 (18)
C27—P1	1.830 (3)	Mo1—O6^i^	1.9293 (18)
C28—C29	1.381 (4)	Mo1—O9	2.3248 (11)
C28—H28	0.9300	Mo2—O9	2.3096 (5)
C33—C38	1.378 (4)	Mo3—O10^i^	1.9238 (18)
C33—C34	1.385 (4)	Mo3—O9	2.3165 (7)
C33—P2	1.827 (2)	Cu1—P2	2.2327 (9)
C29—C30	1.359 (5)	Cu1—P1	2.2866 (10)
C29—H29	0.9300	O9—Mo2^i^	2.3096 (5)
C34—C35	1.388 (4)	O9—Mo3^i^	2.3165 (7)
C34—H34	0.9300	O9—Mo1^i^	2.3248 (11)
			
C2—N1—C6	117.6 (2)	C41—C40—C39	120.9 (3)
C2—N1—Cu1	130.46 (18)	C41—C40—H40	119.5
C6—N1—Cu1	111.25 (16)	C39—C40—H40	119.5
N1—C2—C3	123.5 (3)	C42—C41—C40	120.6 (3)
N1—C2—H2	118.3	C42—C41—H41	119.7
C3—C2—H2	118.3	C40—C41—H41	119.7
C4—C3—C2	119.1 (3)	C43—C42—C41	119.3 (3)
C4—C3—H3	120.4	C43—C42—H42	120.4
C2—C3—H3	120.4	C41—C42—H42	120.4
C3—C4—C5	119.7 (3)	C42—C43—C44	120.8 (3)
C3—C4—H4	120.1	C42—C43—H43	119.6
C5—C4—H4	120.1	C44—C43—H43	119.6
C4—C5—C6	118.0 (3)	C43—C44—C39	120.5 (3)
C4—C5—C7	123.6 (3)	C43—C44—H44	119.8
C6—C5—C7	118.3 (3)	C39—C44—H44	119.8
N1—C6—C5	122.0 (2)	C50—C45—C46	118.3 (2)
N1—C6—C10	117.5 (2)	C50—C45—P2	119.1 (2)
C5—C6—C10	120.5 (2)	C46—C45—P2	122.5 (2)
C8—C7—C5	121.4 (3)	C47—C46—C45	120.5 (3)
C8—C7—H7	119.3	C47—C46—H46	119.8
C5—C7—H7	119.3	C45—C46—H46	119.8
C7—C8—C9	121.8 (3)	C48—C47—C46	120.2 (3)
C7—C8—H8	119.1	C48—C47—H47	119.9
C9—C8—H8	119.1	C46—C47—H47	119.9
C14—C9—C10	117.5 (2)	C49—C48—C47	119.9 (3)
C14—C9—C8	124.0 (3)	C49—C48—H48	120.1
C10—C9—C8	118.5 (3)	C47—C48—H48	120.1
N2—C10—C9	122.4 (2)	C48—C49—C50	120.2 (3)
N2—C10—C6	118.1 (2)	C48—C49—H49	119.9
C9—C10—C6	119.5 (2)	C50—C49—H49	119.9
C12—N2—C10	118.2 (2)	C45—C50—C49	121.0 (3)
C12—N2—Cu1	129.71 (17)	C45—C50—H50	119.5
C10—N2—Cu1	111.05 (16)	C49—C50—H50	119.5
N2—C12—C13	122.8 (3)	Mo3—O1—Mo2	116.14 (9)
N2—C12—H12	118.6	Mo1—O3—Mo2	116.61 (8)
C13—C12—H12	118.6	Mo1^i^—O4—Mo3	116.64 (9)
C14—C13—C12	119.3 (3)	Mo3—O5—Mo1	116.44 (8)
C14—C13—H13	120.3	Mo2—O6—Mo1^i^	116.49 (9)
C12—C13—H13	120.3	Mo2—O10—Mo3^i^	116.62 (9)
C13—C14—C9	119.8 (3)	O7—Mo1—O4^i^	103.95 (9)
C13—C14—H14	120.1	O7—Mo1—O3	103.37 (9)
C9—C14—H14	120.1	O4^i^—Mo1—O3	88.49 (8)
C16—C15—C20	118.8 (2)	O7—Mo1—O6^i^	103.46 (9)
C16—C15—P1	121.08 (19)	O4^i^—Mo1—O6^i^	87.56 (8)
C20—C15—P1	119.35 (19)	O3—Mo1—O6^i^	153.05 (7)
C17—C16—C15	120.0 (2)	O7—Mo1—O5	102.67 (9)
C17—C16—H16	120.0	O4^i^—Mo1—O5	153.37 (7)
C15—C16—H16	120.0	O3—Mo1—O5	86.14 (8)
C18—C17—C16	120.4 (3)	O6^i^—Mo1—O5	85.56 (8)
C18—C17—H17	119.8	O7—Mo1—O9	178.81 (7)
C16—C17—H17	119.8	O4^i^—Mo1—O9	77.24 (6)
C19—C18—C17	119.9 (2)	O3—Mo1—O9	76.61 (6)
C19—C18—H18	120.1	O6^i^—Mo1—O9	76.52 (6)
C17—C18—H18	120.1	O5—Mo1—O9	76.15 (5)
C18—C19—C20	120.0 (3)	O2—Mo2—O10	103.27 (9)
C18—C19—H19	120.0	O2—Mo2—O6	102.58 (9)
C20—C19—H19	120.0	O10—Mo2—O6	87.67 (8)
C19—C20—C15	120.9 (2)	O2—Mo2—O3	103.70 (9)
C19—C20—H20	119.6	O10—Mo2—O3	86.88 (8)
C15—C20—H20	119.6	O6—Mo2—O3	153.71 (7)
C26—C21—C22	118.5 (3)	O2—Mo2—O1	103.00 (9)
C26—C21—P1	118.4 (2)	O10—Mo2—O1	153.73 (7)
C22—C21—P1	123.0 (2)	O6—Mo2—O1	86.97 (8)
C23—C22—C21	120.7 (3)	O3—Mo2—O1	86.63 (8)
C23—C22—H22	119.7	O2—Mo2—O9	179.56 (8)
C21—C22—H22	119.7	O10—Mo2—O9	76.86 (6)
C24—C23—C22	120.1 (3)	O6—Mo2—O9	77.00 (6)
C24—C23—H23	119.9	O3—Mo2—O9	76.72 (6)
C22—C23—H23	119.9	O1—Mo2—O9	76.87 (6)
C25—C24—C23	120.0 (3)	O8—Mo3—O5	104.52 (9)
C25—C24—H24	120.0	O8—Mo3—O1	103.60 (9)
C23—C24—H24	120.0	O5—Mo3—O1	88.27 (8)
C24—C25—C26	120.4 (3)	O8—Mo3—O10^i^	102.87 (9)
C24—C25—H25	119.8	O5—Mo3—O10^i^	87.76 (8)
C26—C25—H25	119.8	O1—Mo3—O10^i^	153.39 (7)
C21—C26—C25	120.3 (3)	O8—Mo3—O4	101.95 (9)
C21—C26—H26	119.8	O5—Mo3—O4	153.53 (7)
C25—C26—H26	119.8	O1—Mo3—O4	85.93 (8)
C32—C27—C28	118.7 (3)	O10^i^—Mo3—O4	85.99 (8)
C32—C27—P1	118.3 (2)	O8—Mo3—O9	178.16 (7)
C28—C27—P1	123.0 (2)	O5—Mo3—O9	77.25 (5)
C29—C28—C27	120.2 (3)	O1—Mo3—O9	76.88 (6)
C29—C28—H28	119.9	O10^i^—Mo3—O9	76.57 (6)
C27—C28—H28	119.9	O4—Mo3—O9	76.28 (6)
C38—C33—C34	118.7 (2)	N1—Cu1—N2	80.24 (8)
C38—C33—P2	123.6 (2)	N1—Cu1—P2	112.06 (6)
C34—C33—P2	117.71 (19)	N2—Cu1—P2	123.26 (6)
C30—C29—C28	120.8 (3)	N1—Cu1—P1	101.36 (7)
C30—C29—H29	119.6	N2—Cu1—P1	99.82 (6)
C28—C29—H29	119.6	P2—Cu1—P1	128.31 (3)
C33—C34—C35	120.3 (3)	C15—P1—C27	104.56 (12)
C33—C34—H34	119.8	C15—P1—C21	103.74 (11)
C35—C34—H34	119.8	C27—P1—C21	103.67 (12)
C36—C35—C34	120.1 (3)	C15—P1—Cu1	106.58 (8)
C36—C35—H35	120.0	C27—P1—Cu1	115.18 (9)
C34—C35—H35	120.0	C21—P1—Cu1	121.40 (9)
C29—C30—C31	120.1 (3)	C45—P2—C33	105.64 (12)
C29—C30—H30A	120.0	C45—P2—C39	102.26 (11)
C31—C30—H30A	120.0	C33—P2—C39	103.70 (11)
C37—C36—C35	120.0 (3)	C45—P2—Cu1	114.30 (9)
C37—C36—H36	120.0	C33—P2—Cu1	115.88 (8)
C35—C36—H36	120.0	C39—P2—Cu1	113.58 (8)
C30—C31—C32	120.0 (3)	Mo2—O9—Mo2^i^	180.0
C30—C31—H31A	120.0	Mo2—O9—Mo3^i^	89.91 (3)
C32—C31—H31A	120.0	Mo2^i^—O9—Mo3^i^	90.09 (3)
C27—C32—C31	120.2 (3)	Mo2—O9—Mo3	90.09 (3)
C27—C32—H32	119.9	Mo2^i^—O9—Mo3	89.91 (3)
C31—C32—H32	119.9	Mo3^i^—O9—Mo3	180.0
C36—C37—C38	120.4 (3)	Mo2—O9—Mo1^i^	89.98 (3)
C36—C37—H37	119.8	Mo2^i^—O9—Mo1^i^	90.02 (3)
C38—C37—H37	119.8	Mo3^i^—O9—Mo1^i^	90.16 (2)
C33—C38—C37	120.6 (3)	Mo3—O9—Mo1^i^	89.84 (2)
C33—C38—H38	119.7	Mo2—O9—Mo1	90.02 (3)
C37—C38—H38	119.7	Mo2^i^—O9—Mo1	89.98 (3)
C40—C39—C44	118.0 (2)	Mo3^i^—O9—Mo1	89.84 (2)
C40—C39—P2	123.7 (2)	Mo3—O9—Mo1	90.16 (2)
C44—C39—P2	118.2 (2)	Mo1^i^—O9—Mo1	180.0
			
C6—N1—C2—C3	0.3 (4)	C12—N2—Cu1—N1	−179.2 (2)
Cu1—N1—C2—C3	−169.11 (19)	C10—N2—Cu1—N1	−11.55 (15)
N1—C2—C3—C4	0.9 (4)	C12—N2—Cu1—P2	70.7 (2)
C2—C3—C4—C5	−0.1 (4)	C10—N2—Cu1—P2	−121.60 (14)
C3—C4—C5—C6	−1.8 (4)	C12—N2—Cu1—P1	−79.3 (2)
C3—C4—C5—C7	178.2 (3)	C10—N2—Cu1—P1	88.41 (15)
C2—N1—C6—C5	−2.3 (3)	C16—C15—P1—C27	32.1 (2)
Cu1—N1—C6—C5	169.06 (18)	C20—C15—P1—C27	−158.2 (2)
C2—N1—C6—C10	178.9 (2)	C16—C15—P1—C21	140.5 (2)
Cu1—N1—C6—C10	−9.7 (3)	C20—C15—P1—C21	−49.9 (2)
C4—C5—C6—N1	3.1 (4)	C16—C15—P1—Cu1	−90.3 (2)
C7—C5—C6—N1	−176.9 (2)	C20—C15—P1—Cu1	79.3 (2)
C4—C5—C6—C10	−178.2 (2)	C32—C27—P1—C15	−104.8 (2)
C7—C5—C6—C10	1.8 (4)	C28—C27—P1—C15	76.0 (2)
C4—C5—C7—C8	178.3 (3)	C32—C27—P1—C21	146.8 (2)
C6—C5—C7—C8	−1.7 (4)	C28—C27—P1—C21	−32.4 (2)
C5—C7—C8—C9	0.2 (4)	C32—C27—P1—Cu1	11.8 (2)
C7—C8—C9—C14	179.5 (3)	C28—C27—P1—Cu1	−167.43 (19)
C7—C8—C9—C10	1.2 (4)	C26—C21—P1—C15	152.6 (2)
C14—C9—C10—N2	−0.8 (3)	C22—C21—P1—C15	−31.3 (3)
C8—C9—C10—N2	177.6 (2)	C26—C21—P1—C27	−98.4 (2)
C14—C9—C10—C6	−179.4 (2)	C22—C21—P1—C27	77.7 (3)
C8—C9—C10—C6	−1.0 (3)	C26—C21—P1—Cu1	33.1 (3)
N1—C6—C10—N2	−0.3 (3)	C22—C21—P1—Cu1	−150.9 (2)
C5—C6—C10—N2	−179.1 (2)	N1—Cu1—P1—C15	27.33 (11)
N1—C6—C10—C9	178.3 (2)	N2—Cu1—P1—C15	−54.57 (10)
C5—C6—C10—C9	−0.5 (3)	P2—Cu1—P1—C15	157.64 (9)
C9—C10—N2—C12	0.8 (3)	N1—Cu1—P1—C27	−88.12 (11)
C6—C10—N2—C12	179.4 (2)	N2—Cu1—P1—C27	−170.03 (10)
C9—C10—N2—Cu1	−168.51 (18)	P2—Cu1—P1—C27	42.18 (9)
C6—C10—N2—Cu1	10.1 (2)	N1—Cu1—P1—C21	145.51 (11)
C10—N2—C12—C13	−0.2 (4)	N2—Cu1—P1—C21	63.60 (12)
Cu1—N2—C12—C13	166.7 (2)	P2—Cu1—P1—C21	−84.19 (11)
N2—C12—C13—C14	−0.2 (4)	C50—C45—P2—C33	110.7 (2)
C12—C13—C14—C9	0.2 (4)	C46—C45—P2—C33	−72.0 (2)
C10—C9—C14—C13	0.4 (4)	C50—C45—P2—C39	−141.0 (2)
C8—C9—C14—C13	−177.9 (3)	C46—C45—P2—C39	36.2 (2)
C20—C15—C16—C17	−0.5 (4)	C50—C45—P2—Cu1	−17.9 (3)
P1—C15—C16—C17	169.2 (2)	C46—C45—P2—Cu1	159.4 (2)
C15—C16—C17—C18	0.4 (4)	C38—C33—P2—C45	7.8 (3)
C16—C17—C18—C19	0.3 (5)	C34—C33—P2—C45	−171.1 (2)
C17—C18—C19—C20	−1.1 (4)	C38—C33—P2—C39	−99.3 (3)
C18—C19—C20—C15	1.0 (4)	C34—C33—P2—C39	81.7 (2)
C16—C15—C20—C19	−0.2 (4)	C38—C33—P2—Cu1	135.5 (2)
P1—C15—C20—C19	−170.1 (2)	C34—C33—P2—Cu1	−43.4 (2)
C26—C21—C22—C23	1.8 (5)	C40—C39—P2—C45	−133.6 (2)
P1—C21—C22—C23	−174.2 (3)	C44—C39—P2—C45	50.0 (2)
C21—C22—C23—C24	−0.2 (5)	C40—C39—P2—C33	−23.9 (2)
C22—C23—C24—C25	−0.4 (6)	C44—C39—P2—C33	159.7 (2)
C23—C24—C25—C26	−0.7 (6)	C40—C39—P2—Cu1	102.7 (2)
C22—C21—C26—C25	−2.9 (5)	C44—C39—P2—Cu1	−73.7 (2)
P1—C21—C26—C25	173.3 (3)	N1—Cu1—P2—C45	−60.99 (11)
C24—C25—C26—C21	2.4 (5)	N2—Cu1—P2—C45	31.70 (12)
C32—C27—C28—C29	−0.1 (4)	P1—Cu1—P2—C45	172.79 (9)
P1—C27—C28—C29	179.1 (2)	N1—Cu1—P2—C33	175.78 (11)
C27—C28—C29—C30	0.5 (5)	N2—Cu1—P2—C33	−91.53 (12)
C38—C33—C34—C35	−0.1 (5)	P1—Cu1—P2—C33	49.56 (10)
P2—C33—C34—C35	179.0 (3)	N1—Cu1—P2—C39	55.85 (11)
C33—C34—C35—C36	−0.7 (5)	N2—Cu1—P2—C39	148.54 (11)
C28—C29—C30—C31	0.1 (5)	P1—Cu1—P2—C39	−70.38 (9)
C34—C35—C36—C37	1.1 (6)	O2—Mo2—O9—Mo2^i^	−7 (100)
C29—C30—C31—C32	−1.0 (5)	O10—Mo2—O9—Mo2^i^	100 (100)
C28—C27—C32—C31	−0.8 (4)	O6—Mo2—O9—Mo2^i^	9 (100)
P1—C27—C32—C31	179.9 (2)	O3—Mo2—O9—Mo2^i^	−170 (100)
C30—C31—C32—C27	1.4 (4)	O1—Mo2—O9—Mo2^i^	−81 (100)
C35—C36—C37—C38	−0.7 (6)	O2—Mo2—O9—Mo3^i^	−106 (10)
C34—C33—C38—C37	0.5 (5)	O10—Mo2—O9—Mo3^i^	1.23 (5)
P2—C33—C38—C37	−178.5 (3)	O6—Mo2—O9—Mo3^i^	−89.40 (6)
C36—C37—C38—C33	−0.1 (6)	O3—Mo2—O9—Mo3^i^	91.10 (6)
C44—C39—C40—C41	0.4 (4)	O1—Mo2—O9—Mo3^i^	−179.29 (5)
P2—C39—C40—C41	−176.0 (2)	O2—Mo2—O9—Mo3	74 (10)
C39—C40—C41—C42	−0.9 (5)	O10—Mo2—O9—Mo3	−178.77 (5)
C40—C41—C42—C43	1.1 (5)	O6—Mo2—O9—Mo3	90.60 (6)
C41—C42—C43—C44	−1.0 (5)	O3—Mo2—O9—Mo3	−88.90 (6)
C42—C43—C44—C39	0.6 (5)	O1—Mo2—O9—Mo3	0.71 (5)
C40—C39—C44—C43	−0.3 (4)	O2—Mo2—O9—Mo1^i^	−16 (10)
P2—C39—C44—C43	176.3 (2)	O10—Mo2—O9—Mo1^i^	91.39 (6)
C50—C45—C46—C47	0.7 (4)	O6—Mo2—O9—Mo1^i^	0.76 (6)
P2—C45—C46—C47	−176.6 (2)	O3—Mo2—O9—Mo1^i^	−178.74 (6)
C45—C46—C47—C48	0.0 (5)	O1—Mo2—O9—Mo1^i^	−89.13 (6)
C46—C47—C48—C49	−0.8 (5)	O2—Mo2—O9—Mo1	164 (32)
C47—C48—C49—C50	0.8 (6)	O10—Mo2—O9—Mo1	−88.61 (6)
C46—C45—C50—C49	−0.6 (5)	O6—Mo2—O9—Mo1	−179.24 (6)
P2—C45—C50—C49	176.8 (3)	O3—Mo2—O9—Mo1	1.26 (6)
C48—C49—C50—C45	−0.1 (6)	O1—Mo2—O9—Mo1	90.87 (6)
Mo2—O3—Mo1—O7	−179.51 (10)	O8—Mo3—O9—Mo2	−106 (2)
Mo2—O3—Mo1—O4^i^	−75.53 (10)	O5—Mo3—O9—Mo2	90.49 (6)
Mo2—O3—Mo1—O6^i^	6.1 (2)	O1—Mo3—O9—Mo2	−0.71 (5)
Mo2—O3—Mo1—O5	78.37 (10)	O10^i^—Mo3—O9—Mo2	−178.77 (5)
Mo2—O3—Mo1—O9	1.70 (8)	O4—Mo3—O9—Mo2	−89.67 (6)
Mo3—O5—Mo1—O7	−179.37 (10)	O8—Mo3—O9—Mo2^i^	74 (2)
Mo3—O5—Mo1—O4^i^	2.3 (2)	O5—Mo3—O9—Mo2^i^	−89.51 (6)
Mo3—O5—Mo1—O3	−76.52 (10)	O1—Mo3—O9—Mo2^i^	179.29 (5)
Mo3—O5—Mo1—O6^i^	77.82 (10)	O10^i^—Mo3—O9—Mo2^i^	1.23 (5)
Mo3—O5—Mo1—O9	0.62 (7)	O4—Mo3—O9—Mo2^i^	90.33 (6)
Mo3^i^—O10—Mo2—O2	177.90 (10)	O8—Mo3—O9—Mo3^i^	61 (100)
Mo3^i^—O10—Mo2—O6	75.53 (10)	O5—Mo3—O9—Mo3^i^	−102 (100)
Mo3^i^—O10—Mo2—O3	−78.75 (10)	O1—Mo3—O9—Mo3^i^	167 (100)
Mo3^i^—O10—Mo2—O1	−2.8 (2)	O10^i^—Mo3—O9—Mo3^i^	−11 (100)
Mo3^i^—O10—Mo2—O9	−1.66 (7)	O4—Mo3—O9—Mo3^i^	78 (100)
Mo1^i^—O6—Mo2—O2	178.84 (10)	O8—Mo3—O9—Mo1^i^	−16 (2)
Mo1^i^—O6—Mo2—O10	−78.08 (10)	O5—Mo3—O9—Mo1^i^	−179.53 (6)
Mo1^i^—O6—Mo2—O3	0.1 (2)	O1—Mo3—O9—Mo1^i^	89.27 (6)
Mo1^i^—O6—Mo2—O1	76.19 (10)	O10^i^—Mo3—O9—Mo1^i^	−88.79 (6)
Mo1^i^—O6—Mo2—O9	−1.03 (7)	O4—Mo3—O9—Mo1^i^	0.30 (6)
Mo1—O3—Mo2—O2	178.42 (10)	O8—Mo3—O9—Mo1	164 (2)
Mo1—O3—Mo2—O10	75.52 (10)	O5—Mo3—O9—Mo1	0.47 (6)
Mo1—O3—Mo2—O6	−2.8 (2)	O1—Mo3—O9—Mo1	−90.73 (6)
Mo1—O3—Mo2—O1	−79.01 (10)	O10^i^—Mo3—O9—Mo1	91.21 (6)
Mo1—O3—Mo2—O9	−1.71 (8)	O4—Mo3—O9—Mo1	−179.70 (6)
Mo3—O1—Mo2—O2	179.49 (10)	O7—Mo1—O9—Mo2	−90 (4)
Mo3—O1—Mo2—O10	0.2 (2)	O4^i^—Mo1—O9—Mo2	90.22 (6)
Mo3—O1—Mo2—O6	−78.30 (10)	O3—Mo1—O9—Mo2	−1.27 (6)
Mo3—O1—Mo2—O3	76.19 (10)	O6^i^—Mo1—O9—Mo2	−179.24 (5)
Mo3—O1—Mo2—O9	−0.95 (7)	O5—Mo1—O9—Mo2	−90.55 (6)
Mo1—O5—Mo3—O8	179.93 (10)	O7—Mo1—O9—Mo2^i^	90 (4)
Mo1—O5—Mo3—O1	76.32 (10)	O4^i^—Mo1—O9—Mo2^i^	−89.78 (6)
Mo1—O5—Mo3—O10^i^	−77.36 (10)	O3—Mo1—O9—Mo2^i^	178.73 (6)
Mo1—O5—Mo3—O4	−1.0 (2)	O6^i^—Mo1—O9—Mo2^i^	0.76 (5)
Mo1—O5—Mo3—O9	−0.62 (7)	O5—Mo1—O9—Mo2^i^	89.45 (6)
Mo2—O1—Mo3—O8	179.12 (10)	O7—Mo1—O9—Mo3^i^	180 (100)
Mo2—O1—Mo3—O5	−76.36 (10)	O4^i^—Mo1—O9—Mo3^i^	0.31 (6)
Mo2—O1—Mo3—O10^i^	5.2 (2)	O3—Mo1—O9—Mo3^i^	−91.18 (6)
Mo2—O1—Mo3—O4	77.79 (10)	O6^i^—Mo1—O9—Mo3^i^	90.85 (6)
Mo2—O1—Mo3—O9	0.95 (7)	O5—Mo1—O9—Mo3^i^	179.54 (5)
Mo1^i^—O4—Mo3—O8	179.05 (11)	O7—Mo1—O9—Mo3	0 (4)
Mo1^i^—O4—Mo3—O5	−0.1 (2)	O4^i^—Mo1—O9—Mo3	−179.69 (6)
Mo1^i^—O4—Mo3—O1	−77.89 (11)	O3—Mo1—O9—Mo3	88.82 (6)
Mo1^i^—O4—Mo3—O10^i^	76.73 (11)	O6^i^—Mo1—O9—Mo3	−89.15 (6)
Mo1^i^—O4—Mo3—O9	−0.42 (8)	O5—Mo1—O9—Mo3	−0.46 (5)
C2—N1—Cu1—N2	−178.7 (2)	O7—Mo1—O9—Mo1^i^	86 (100)
C6—N1—Cu1—N2	11.37 (15)	O4^i^—Mo1—O9—Mo1^i^	−93 (100)
C2—N1—Cu1—P2	−56.6 (2)	O3—Mo1—O9—Mo1^i^	175 (100)
C6—N1—Cu1—P2	133.43 (14)	O6^i^—Mo1—O9—Mo1^i^	−3 (100)
C2—N1—Cu1—P1	83.2 (2)	O5—Mo1—O9—Mo1^i^	86 (100)
C6—N1—Cu1—P1	−86.79 (16)		

Symmetry code: (i) −*x*+1, −*y*+1, −*z*+1.

### Hydrogen-bond geometry (Å, º)

Cg8 is the centroid of the C33–C38 ring.

**Table:**

*D*—H···*A*	*D*—H	H···*A*	*D*···*A*	*D*—H···*A*
C19—H19···O3^ii^	0.93	2.60	3.446 (3)	152
C29—H29···O7^iii^	0.93	2.55	3.443 (4)	161
C30—H30*A*···O8^iv^	0.93	2.41	3.257 (5)	152
C44—H44···O2	0.93	2.51	3.206 (4)	132
C49—H49···*Cg*8^v^	0.93	2.97	3.782 (5)	147

Symmetry codes: (ii) *x*+1, *y*, *z*; (iii) *x*+1, *y*+1, *z*; (iv) −*x*+1, −*y*+2, −*z*+1; (v) −*x*+1, −*y*+1, −*z*.
